# Evaluating the Impact and Financial Implications of Immediate versus Delayed Stenting Strategies in High Thrombus Burden Acute Myocardial Infarction: A Propensity Score-Matched Analysis

**DOI:** 10.31083/j.rcm2510381

**Published:** 2024-10-24

**Authors:** Bin Xie, Jilin Li, Weiwen Li, Ying Lin, Huaiwen Wang

**Affiliations:** ^1^Department of Cardiovascular, The Second Affiliated Hospital of Medical College of Shantou University, 515000 Shantou, Guangdong, China

**Keywords:** immediate stenting, delayed stenting, acute myocardial infarction

## Abstract

**Background:**

The efficacy of delayed stenting strategies in the management of high thrombus burden acute myocardial infarction remains uncertain. We aimed to compare the therapeutic effects and financial implications of immediate and delayed stenting strategies in patients with acute myocardial infarction and high thrombus burden treated at our institution.

**Methods:**

This was a retrospective analysis of 158 patients who underwent intracoronary thrombus aspiration for acute ST-elevation myocardial infarction (STEMI) at the Second Affiliated Hospital of Shantou University Medical College between 2013 and 2023. Patients were divided into two groups: immediate stenting (immediate group; n = 101) and delayed stenting (delayed group; n = 57), based on the timing of the stenting procedure. Propensity score matching was performed to minimize confounding bias. Therapeutic effects and cost of treatment were compared between the two groups.

**Results:**

After propensity score matching (n = 52 for each group), there were no significant differences in terms of baseline clinical characteristics, characteristics of vascular lesions (number of diseased vessels, culprit vessels, thrombolysis in myocardial infarction (TIMI) thrombus grade, proximal coronary artery lesion), the incidence of no-reflow/slow flow during the first surgery, or the use of antiplatelet drugs, intraprocedural anticoagulants, intracoronary drugs, and tirofiban. There were no significant between-group differences in terms of in-hospital all-cause mortality, in-hospital major adverse cardiovascular events, or hospitalization costs. However, peak creatine kinase-myocardial band (CK-MB) levels were significantly lower in the delayed group.

**Conclusions:**

For patients with STEMI undergoing emergency thrombus aspiration, a delayed stenting strategy appears to be non-inferior to immediate stenting strategy in terms of clinical efficacy and hospitalization costs, and may reduce the extent of myocardial injury. Delayed stenting strategy may allow for a more individualized surgical approach based on assessment of thrombus burden and lesion complexity.

## 1. Introduction 

The development of chest pain centers across China has helped improve the care for patients with acute myocardial infarction (MI). Prompt and comprehensive revascularization is crucial for enhancing outcomes in patients with acute ST-segment elevation myocardial infarction (STEMI) [1, 2, 3, 4]. Immediately restoring coronary artery patency through the first percutaneous coronary intervention (PCI) is the gold standard for treating STEMI [5]. However, despite rapid reperfusion, 10%–40% of patients still experience microcirculatory dysfunction after vascular reconstruction [6, 7], a condition referred to as “no-reflow” or “slow flow”, indicating suboptimal myocardial reperfusion [8]. Distal embolization (DE) of atherosclerotic plaque fragments during manipulation of the culprit vessel is a major risk associated with immediate stenting [9, 10]. Contemporary research comparing routine stenting to balloon angioplasty alone shows a decrease in ischemia-driven revascularization for STEMI patients with the latter approach, without impacting mortality or re-infarction rates [11, 12, 13].

For these reasons, a two-step treatment for high thrombus load lesions, including quick mechanical reflow via balloon expansion and/or thrombectomy with subsequent stent implantation delayed for several days, has been introduced [14]. This delayed approach allows for the reduction of thrombus burden with anticoagulant and antiplatelet medications, thereby reducing the risk of stent-related DE and no-reflow [14, 15]. Additionally, the removal of intraluminal thrombi and the gradual resolution of vascular reactivity may help reveal the true lumen diameter, facilitating optimal stent sizing and potentially reducing the risk of stent malapposition, under-expansion, or poor apposition [16, 17]. Several studies in [18] have demonstrated the safety and feasibility of delayed stent placement, and an initial meta-analysis has affirmed its angiographic advantages without impacting the incidence of major adverse cardiovascular events (MACEs). Recent randomized controlled trials (RCTs) have provided additional evidence regarding this approach, although results on clinical efficacy remain mixed [19, 20]. In a large-scale randomized trial (n = 1215), delayed stenting did not significantly improve primary clinical outcomes [21]. Additionally, delayed stenting necessitates a second procedure, escalating the financial burden on patients due to increased hospitalization fees, surgical costs, and costs of medication and consumables, aspects not extensively examined in previous studies. Therefore, we aimed to retrospectively assess the clinical efficacy and financial benefits of immediate versus delayed stenting strategies for patients with high thrombus burden STEMI.

## 2. Materials and Methods

### 2.1 Research Subjects

Patients with acute STEMI treated at the Second Affiliated Hospital of Shantou University Medical College between 2013 and 2023 were retrospectively reviewed. All patients met the STEMI diagnostic criteria outlined in the “Acute ST-Segment Elevation Myocardial Infarction Diagnosis and Treatment Guidelines (2019)” and underwent intracoronary thrombus aspiration. The inclusion criteria were: (1) onset of symptoms ≤24 hours; (2) age >18 years. The exclusion criteria were: (1) a history of bleeding, trauma, or organ surgery within the last month or active gastrointestinal ulcers; (2) coexisting conditions such as aortic aneurysm dissection, infective endocarditis, severe cardiogenic shock, severe left heart failure, or intracranial tumors; (3) a history of cerebral hemorrhage, subarachnoid hemorrhage, or stroke; (4) concurrent hematologic, hemorrhagic diseases or bleeding tendencies; (5) severe liver or kidney dysfunction; (6) pregnancy.

Patients were divided into an immediate stent implantation group (immediate group) and a deferred angiography stent implantation group (delayed group) based on the treatment strategy implemented. The choice of treatment strategy was at the discretion of the operating clinician. The deferred stenting strategy entailed a 7–9 day delay in angiography, and this group included 19 cases where patients and their families refused scheduled coronary angiography and 7 cases where angiography was performed without subsequent stent implantation. The immediate group comprised 101 cases, while the delayed group included 57 cases. This retrospective study was approved by the hospital’s ethics committee.

### 2.2 Treatment Methods

All patients underwent emergency coronary angiography, and all presented with high thrombus burden lesions. Preprocedural dual antiplatelet loading therapy was administered to all patients (aspirin 300 mg + clopidogrel 300 mg or aspirin 300 mg + ticagrelor 180 mg). Intraprocedural coronary thrombosuction was performed in all cases. The intraprocedural anticoagulation regimen consisted of heparin (100 U/kg) or bivalirudin (0.75 mg/kg bolus followed by 1.75 mg/kg/h maintenance infusion, continued for 4 hours postoperatively). During the procedure, tirofiban, adenosine, nitroprusside, nitroglycerin, and pro-urokinase were administered as needed. The decision to implant a stent was based on the surgeon’s experience, considering both immediate and delayed stenting strategies, and follow-up imaging was used to exclude non-high thrombus burden.

### 2.3 Data Collection

Data pertaining to sex, age, and traditional risk factors for coronary artery disease, including hypertension, diabetes, history of smoking, and history of PCI were retrieved. Additionally, the following diagnostic information and lesion characteristics were recorded: the number of diseased vessels, the culprit vessels, the thrombolysis in myocardial infarction (TIMI) thrombus grade, and the presence of proximal coronary artery lesions. Clinical indicators measured included low-density lipoprotein cholesterol (LDL-C), creatinine (Cr), C-reactive protein (CRP), peak creatine kinase-myocardial band (CK-MB), and left ventricular ejection fraction (LVEF). The details of the assessment of the initial postprocedural TIMI flow grade. Intraprocedural management focused on antiplatelet and anticoagulation strategies, particularly in instances of no-reflow or slow flow during the procedure. The administration of intracoronary drugs and tirofiban was recorded. For patients scheduled for delayed stenting, the postprocedural TIMI flow grade was carefully evaluated. Post-emergency PCI variables included the continuation of tirofiban therapy and the introduction of low-molecular-weight heparin. Furthermore, the planning and outcomes of follow-up coronary angiography and delayed stent placement were thoroughly reviewed.

### 2.4 Efficacy Evaluation and Hospitalization Costs

(1) In-hospital all-cause mortality; (2) In-hospital MACE, including recurrent MI, stroke, cardiovascular death, malignant arrhythmias, acute heart failure, and cardiogenic shock; (3) Hospitalization costs, including total costs, surgical fees, material fees, and medicine costs.

### 2.5 Statistical Methods

Statistical analysis was conducted using R software (version 4.4.0; R Foundation for Statistical Computing, Vienna, Austria; https://www.r-project.org/). Propensity score matching was performed to improve comparability between the immediate and delayed groups. The variables included in the propensity score model were age, hypertension, diabetes, history of smoking, number of diseased vessels, culprit vessels, and TIMI thrombus grade. A caliper width of 0.2 was used for matching. The normality of continuous variables was assessed using the Shapiro-Wilk test. Normally distributed continuous variables were expressed as mean ± standard deviation (SD), and between-group differences were assessed using the Student’s *t*-test. Non-normally distributed continuous variables were expressed as median (interquartile range) and compared using the Mann-Whitney U test. Categorical variables were expressed as frequency (percentage) (n [%]) and between-group differences were assessed using the Chi-square test or Fisher’s exact test. *p*-values < 0.05 were considered indicative of statistical significance. The impact of immediate versus delayed stenting on in-hospital all-cause mortality was analyzed using Firth’s logistic regression model, adjusting for multiple covariates.

## 3. Results

**Fig. 1.**
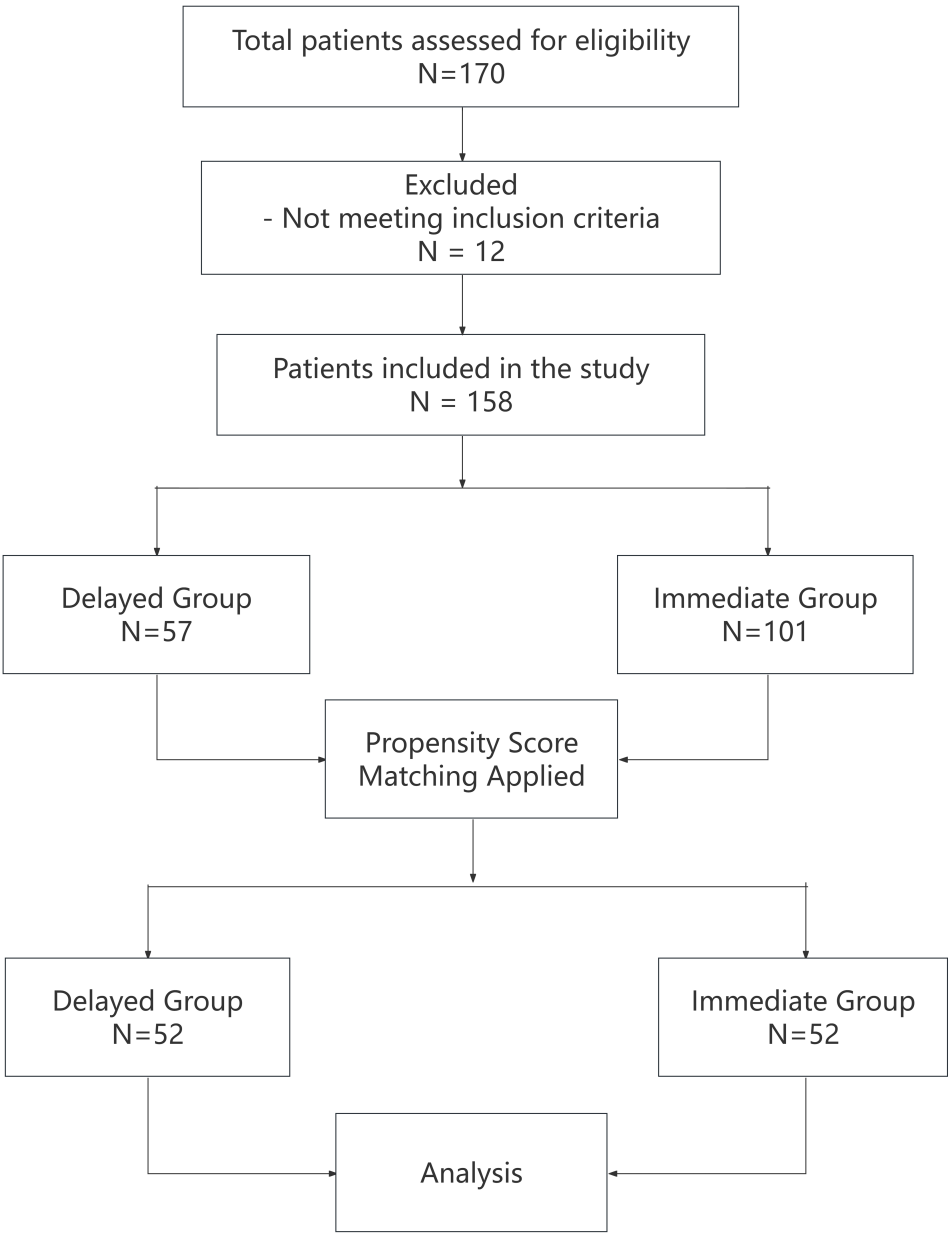
**Study flowchart**.

A total of 170 patients were assessed for eligibility. Of these, 12 patients were excluded based on the exclusion criteria, resulting in 158 patients being included in the study. These patients were stratified according to the surgical strategy into either the Delayed group (n = 57) or the Immediate group (n = 101). To adjust for potential baseline differences between the groups, propensity score matching (PSM) was conducted. Following matching, each group consisted of 52 patients, resulting in a final analysis cohort of 104 patients (Fig. 1).

### 3.1 Comparison of Baseline Characteristics and Clinical Features between the Immediate and Delayed Groups

There were significant differences between the immediate and delayed groups in terms of age and creatinine levels (*p* < 0.05) (Table 1). However, in the propensity score matched cohort, there were no significant between-group differences with respect to sex, age, body mass index (BMI), hypertension, diabetes, smoking, history of PCI, time from onset to hospital visit, characteristics of vascular lesions (number of diseased vessels, culprit vessels, TIMI thrombus grade, proximal coronary artery lesion), and key laboratory markers (creatinine, LDL-C, CRP) (*p* > 0.05 for all; Table 2). This indicated the comparability between the propensity score-matched cohorts.

**Table 1.** **Comparison of baseline characteristics before propensity score matching**.

Indicator		Delayed group (n = 57)	Immediate group (n = 101)	*p* value
Male		51 (89.5)	89 (88.1)	1.000
Age		54.44 ± 12.85	59.20 ± 12.43	0.024
BMI		24.87 ± 3.53	23.79 ± 3.35	0.059
Hypertension		22 (38.6)	53 (52.5)	0.131
Diabetes		16 (28.1)	31 (30.7)	0.869
Long-term smoking history		44 (77.2)	75 (74.3)	0.827
History of PCI		2 (3.5)	1 (1.0)	0.295^$^
Number of diseased vessels				0.092
	1	29 (50.9)	43 (42.6)	
	2	11 (19.3)	36 (35.6)	
	3	17 (29.8)	22 (21.8)	
Culprit vessels				0.054^$^
	LM	13 (22.8)	41 (40.6)	
	LAD	2 (3.5)	3 (3.0)	
	LCX	0 (0.0)	2 (2.0)	
	RCA	42 (73.7)	55 (54.5)	
TIMI thrombus grade				1.000
	Grade 4	8 (14)	13 (12.9)	
	Grade 5	49 (86.0)	88 (87.1)	
Proximal coronary artery lesion		39 (68.4)	67 (66.3)	0.927
Time from onset to hospital visit (h)		4.00 (2.00–8.00)	4.00 (2.00–6.00)	0.498*
Creatinine (µmol/L)		92.00 (85.50–124.70)	86.50 (78.60–107.40)	0.035*
LDL-C (mmol/L)		3.47 ± 1.14	3.36 ± 1.00	0.528
High-sensitivity CRP (mg/L)		9.09 (3.47–30.81)	9.88 (3.63–24.63)	0.631*

*Mann-Whitney U test; $ Fisher’s Exact Test. LM, left main coronary artery; LAD, left anterior descending artery; LCX, left circumflex artery; RCA, right coronary artery; BMI, body mass index; PCI, percutaneous coronary intervention; TIMI, thrombolysis in myocardial infarction; LDL-C, low-density lipoprotein cholesterol; CRP, C-reactive protein.

**Table 2.** **Comparison of baseline characteristics after propensity score matching**.

Indicator		Delayed group (n = 52)	Immediate group (n = 52)	*p* value
Male		46 (88.5)	47 (90.4)	1.000
Age		55.98 ± 12.11	57.69 ± 12.56	0.481
BMI		24.78 ± 3.60	23.77 ± 3.16	0.131
Hypertension		22 (42.3)	22 (42.3)	1.000
Diabetes		16 (30.8)	12 (23.1)	0.507
Long-term smoking history		41 (78.8)	39 (75.0)	0.816
History of PCI		2 (3.8)	0 (0.0)	0.495^$^
Number of diseased vessels				0.838
	1	25 (48.1)	22 (42.3)	
	2	11 (21.2)	12 (23.1)	
	3	16 (30.8)	18 (34.6)	
Culprit vessels				1.000^$^
	LAD	12 (23.1)	12 (23.1)	
	LCX	2 (3.8)	1 (1.9)	
	RCA	38 (73.1)	39 (75.0)	
TIMI thrombus grade				1.000
	Grade 4	8 (15.4)	9 (17.3)	
	Grade 5	44 (84.6)	43 (82.7)	
Proximal coronary artery lesion		35 (67.3)	33 (63.5)	0.837
Time from onset to hospital visit (h)		4.00 (2.00–8.00)	3.00 (2.00–4.25)	0.188*
Creatinine (µmol/L)		94.90 (87.0–130.1)	86.60 (77.8–121.17)	0.113*
LDL-C (mmol/L)		3.33 (2.81–4.37)	3.25 (2.62–4.03)	0.543*
High-sensitivity CRP (mg/L)		8.67 (3.82–31.42)	9.48 (3.68–16.73)	0.644*

*Mann-Whitney U test; $ Fisher’s Exact Test. LAD, left anterior descending artery; LCX, left circumflex artery; RCA, right coronary artery; BMI, body mass index; PCI, percutaneous coronary intervention; TIMI, thrombolysis in myocardial infarction; LDL-C, low-density lipoprotein cholesterol; CRP, C-reactive protein.

There were no significant differences between the immediate and delayed groups in terms of post-dilation conditions and LVEF (*p* > 0.05; Table 3). The elective angiography rate during hospitalization in the delayed group was 50%, with the degree of vascular stenosis in elective angiography cases being 78.46% (70.85%, 86.08%). There was no elective angiography in the immediate group, and significant differences were observed in the circumstances of elective angiography between the two groups (*p* < 0.001). The peak CK-MB levels were significantly higher in the immediate group compared to the delayed group. The median peak CK-MB level in the immediate group was 273.5 U/L (interquartile range: 174.5–448.5), while in the delayed group it was 194 U/L (interquartile range: 108.3–280.3) (*p* = 0.006).

**Table 3.** **Comparison of clinical characteristics between the immediate and delayed groups**.

Indicator		Delayed group (n = 52)	Immediate group (n = 52)	$\chi^{2}$/Z	*p* value
Elective angiography (during hospitalization)				-	<0.001^$^
	Yes	26 (50)	0 (0)		
	No	26 (50)	52 (100)		
Post-dilation				1.477	0.224
	Yes	16 (30.8)	23 (44.2)		
	No	36 (69.2)	29 (55.8)		
Peak CK-MB (U/L)		194 (108.3–280.3)	273.5 (174.5–448.5)	2.74	0.006*
LVEF (%)		57.50 (52.75–61.00)	57.00 (50.00–61.25)	–0.319	0.752*

Data presented as frequency (percentage); *Mann-Whitney U test; $ Fisher’s Exact Test. CK-MB, creatine kinase-myocardial band; LVEF, left ventricular ejection fraction.

### 3.2 Comparative Analysis of Drug Administration in Immediate and Delayed Groups

There were no significant differences between the immediate and delayed groups regarding the use of antiplatelet medications, preprocedural anticoagulants, intracoronary drugs, and tirofiban (*p* > 0.05; Table 4). The immediate group had a significantly lower usage rate of anticoagulants post-surgery than the delayed group (69.2% vs 90.4%; χ^2^ = 5.967, *p* = 0.015).

**Table 4.** **Comparison of medication use between the immediate and delayed groups**.

Indicator		Delayed group (n = 52)	Immediate group (n = 52)	$\chi^{2}$	*p* value
Antiplatelet drugs				0.000	1
	Clopidogrel	15 (28.8)	15 (28.8)		
	Ticagrelor	37 (71.2)	37 (71.2)		
Anticoagulants during surgery				0.000	1
	Bivalirudin	15 (28.8)	15 (28.8)		
	Heparin	37 (71.2)	37 (71.2)		
Intracoronary medications				-	0.931*
	Urokinase	1 (1.9)	0 (0.0)		
	Adenosine	1 (1.9)	0 (0.0)		
	Sodium nitroprusside	8 (15.4)	7 (13.5)		
	Nitroglycerin	7 (13.5)	8 (15.4)		
	None	35 (67.3)	37 (71.2)		
Tirofiban				2.010	0.156
	Yes	44 (84.6)	37 (71.2)		
	No	8 (15.4)	15 (28.8)		
Postprocedural anticoagulants				5.967	0.015
	Yes	47 (90.4)	36 (69.2)		
	No	5 (9.6)	16 (30.8)		

Data presented as frequency (percentage); *Fisher’s exact test.

### 3.3 Comparison of Immediate and Delayed Groups in Terms of Therapeutic Efficacy Evaluation Indicators

There was no significant difference in the initial postprocedural TIMI flow grading between the delayed and immediate groups (*p* = 0.092; Table 5). A significant between-group difference was observed in the distribution of implanted stent numbers (*p* < 0.001). However, there was no significant between-group difference in the occurrence of no-reflow and slow flow ($\chi^{2}$ = 3.656, *p* = 0.056).

**Table 5.** **Comparison of efficacy evaluation indicators between the immediate and delayed groups**.

Indicator		Delayed group (n = 52)	Immediate group (n = 52)	$\chi^{2}$	*p* value
Initial postprocedural TIMI flow, n (%)				-	0.092*
	Grade 0	0 (0.0)	1 (1.9)		
	Grade 1	1 (1.9)	5 (9.6)		
	Grade 2	3 (5.8)	6 (11.5)		
	Grade 3	48 (92.3)	40 (76.9)		
No reflow or slow flow, n (%)				3.656	0.056
	Yes	21 (40.4)	11 (21.2)		
	No	31 (59.6)	41 (78.8)		
Number of stents implanted, n (%)				-	<0.001*
	0	30 (57.7%)	0 (0.0%)		
	1	15 (28.8%)	39 (75.0%)		
	2	5 (9.6%)	13 (25.0%)		
	3	1 (1.9%)	0 (0.0%)		
	4	0 (0.0%)	0 (0.0%)		
	5	1 (1.9%)	0 (0.0%)		

Data presented as frequency (percentage); *Fisher’s exact test. TIMI, thrombolysis in myocardial infarction.

### 3.4 Prognostic Evaluation Indicators for Immediate and Delayed Groups

The in-hospital all-cause mortality rate was identical in both groups, with 2 patients (3.85%) in each group succumbing. Similarly, the incidence of in-hospital MACE was the same in both groups, with 6 patients (11.54%) affected in each group. Stroke occurred in 0 patients (0.00%) in the delayed group and 1 patient (1.92%) in the immediate group. Cardiovascular death, malignant arrhythmias, and cardiogenic shock were each reported in 2 (3.85%), 1 (1.92%), and 1 (1.92%) patients respectively in both groups. Acute heart failure was seen in 2 patients (3.85%) in the delayed group and 1 patient (1.92%) in the immediate group. Regarding major bleeding events, 2 patients in the delayed group experienced bleeding, while 1 patient in the immediate group experienced bleeding.

The results indicated no significant difference in in-hospital all-cause mortality between the delayed and immediate stenting strategies (odds ratio (OR): 0.745, 95% confidence interval (CI): 0.115–4.623, *p* = 0.741) (Table 6). Additionally, age, hypertension, diabetes, smoking history, culprit vessels, and TIMI thrombus grade did not significantly affect in-hospital all-cause mortality. The impact of diabetes approached significance (OR: 4.757, 95% CI: 0.710–49.255, *p* = 0.108).

**Table 6.** **Firth’s logistic regression on in-hospital all-cause mortality adjusting for multiple covariates**.

Indicator		OR (95% CI)	*p*-value
Stenting strategy		0.745 (0.115, 4.623)	0.741
	Age	0.997 (0.906, 1.095)	0.952
	Hypertension	0.884 (0.124, 5.930)	0.895
	Diabetes	4.757 (0.710, 49.255)	0.108
	Long-term smoking history	0.911 (0.126, 10.287)	0.930
	Culprit vessels (LCX vs LAD)	5.842 (0.030, 1157.735)	0.414
	Culprit vessels (RCA vs LAD)	1.592 (0.120, 214.227)	0.752
	TIMI thrombus grade	1.959 (0.201, 256.158)	0.623
Major bleeding		2.040 (0.179, 23.217)	0.566

OR, odds ratio; CI, confidence interval; LAD, left anterior descending artery; LCX, left circumflex artery; RCA, right coronary artery; TIMI, thrombolysis in myocardial infarction.

### 3.5 Comparison of Medical Expenses between Immediate and Delayed Groups

The comparison of medical costs between the immediate and delayed groups is summarized in Table 7. There were no significant differences in total cost, surgical fees, or material fees between the two groups. However, the delayed group had significantly higher medicine costs compared to the immediate group (*p* = 0.023).

**Table 7.** **Comparison of medical costs and hospital day between the immediate and delayed groups**.

Indicator	Delayed group (n = 52)	Immediate group (n = 52)	Z*	*p*
Total cost ($)	6059.88 (4824.02–8843.16)	6160.31 (4542.93–7638.91)	0.806	0.442
Surgical fees ($)	1634.95 (1087.47–2202.84)	1402.07 (1311.09–1605.77)	0.735	0.465
Material fees ($)	2373.10 (1654.28–3307.26)	2512.26 (1480.93–3620.42)	–0.384	0.704
Medicine costs ($)	687.15 (484.45–941.30)	481.99 (350.31–852.93)	2.282	0.023
Hospital stay (days)	10.00 (8.00–12.00)	8.50 (7.00–10.00)	–2.318	0.020

Data presented as median (interquartile range); *Mann-Whitney U test; $, United States dollars.

## 4. Discussion

Early reperfusion of the infarcted artery and restoration of myocardial perfusion are critical in treating STEMI patients [22]. PCI combined with stent implantation is the conventional gold standard treatment for STEMI patients [23]. However, nearly one-third of patients experience suboptimal myocardial reperfusion after stent implantation in the epicardial coronary artery segment [24]. This condition, known as slow flow or no-reflow, is attributed to microvascular damage or embolism. In this context, stent implantation, a routine procedure, is associated with DE from the fragmentation of most atherosclerotic thromboses, leading to the majority of microvascular injuries [25]. Given these challenges, delayed stent implantation has been proposed as an alternative to the conventional immediate stent implantation procedure to reduce DE in STEMI patients.

This study retrospectively analyzed the different treatment strategies for acute STEMI patients after thrombectomy at our hospital over the past decade. We employed propensity score matching to match the immediate and delayed stent implantation groups to minimize confounding and selection bias. After matching, there were no significant differences between the delayed group and the immediate group in terms of sex, age, BMI, hypertension, diabetes, long-term smoking history, history of PCI, time from onset to hospital visit, creatinine levels, LDL-C levels, and high-sensitivity CRP levels (*p* > 0.05). This indicated the comparability between the two groups. Furthermore, there was no significant difference in the number of vascular lesions, culprit vessels, TIMI thrombus grade, proximal coronary artery lesion, the incidence of no-reflow/slow flow during the first operation, antiplatelet drugs, anticoagulants during surgery, intracoronary drugs, tirofiban, and initial postprocedural TIMI flow grade. This may suggest that the timing of intervention does not significantly influence these particular metrics. The significantly lower peak CK-MB levels observed in the delayed group in our study suggests that delayed stenting might have some advantage in reducing myocardial injury. This is inconsistent with the results of the DANAMI-3-DEFER substudy [26], which indicated that routine delayed stenting did not reduce infarct size. Further large-scale studies are needed to confirm our findings.

However, regarding clinical outcomes, there was no significant difference in the incidence of adverse reactions between the two groups. This suggests that delayed stenting did not confer a significant advantage over immediate stenting in this study. This outcome might also be related to the effects of thrombus aspiration and the residual thrombus burden during the procedure in the immediate stenting group. Similar results were reported by Belle *et al*. [20], indicating that the delayed stent implantation strategy is not superior to the immediate stent implantation strategy. A systematic review by Freixa *et al*. [18] found that while the delayed stenting strategy provides superior angiographic outcomes, it has no significant impact on the incidence of MACEs, which is consistent with some of the findings of the present study.

In this study, the decision to adopt a delayed stenting strategy for the delayed group was based on the operator’s experience. It was observed that a proportion of patients in the delayed stenting strategy did not receive follow-up angiography and stent insertion, likely due to personal reasons or financial constraints. Thus, delayed stenting may lead to a missed opportunity for a secondary PCI, potentially increasing the incidence of MACEs in the long term. Among the patients in the delayed group who underwent follow-up angiography, none of the cases had re-occlusion of the vessels. This finding suggests that rigorous antithrombotic and antiplatelet therapy is relatively safe during the wait for a second surgery in the delayed strategy. However, intensive anticoagulation therapy increases the risk of significant bleeding. In contrast, the frequency of anticoagulation therapy in the immediate stenting group was significantly lower than in the delayed group. Remarkably, in this study, there were no cases of postprocedural acute in-stent thrombosis leading to re-infarction, indicating that the use of postprocedural anticoagulants can be reduced in the immediate group without increasing the risk of re-infarction due to acute in-stent thrombosis. The in-hospital all-cause mortality and in-hospital MACE were not significantly different between the two groups. According to a meta-analysis by Sun *et al*. [27] (8 studies with 744 patients), delayed stenting offers benefits in terms of reduced incidence of MACE (OR: 0.48, 95% CI: 0.25–0.94, *p* = 0.03) compared to immediate stenting, with no significant difference noted in bleeding events (OR: 1.76, 95% CI: 0.40–7.66, *p* = 0.45). However, in the present study, the immediate group did not show a higher incidence of MACE, possibly due to the short observation period for in-hospital MACEs.

Both treatment costs and length of hospital stay are significant patient concerns, and this study found no significant differences between the two groups in terms of total costs, surgical fees, and material costs. Consistent with the study by Luo *et al*. [28], which reported no significant difference in hospitalization costs between the immediate and delayed stent implantation groups (Immediate group: $9789 ± 10,532, Delayed group: $10,321 ± 7846, *p* = 0.74). However, there were significant differences in the length of hospital stay and medication costs. This outcome suggests that delayed PCI does not increase the overall economic burden, but it does lead to higher medication costs and longer hospital stays. Clinically, delayed stenting allows for a more detailed assessment of the patient’s condition before the second surgery, helping to circumvent pitfalls during stent placement and potentially minimizing treatment costs.

Some limitations of this study should be acknowledged. Firstly, the retrospective observational design presents challenges in eliminating selection bias. To mitigate this, propensity score matching was employed to improve comparability between the two groups in terms of baseline characteristics and other potential confounding factors. Secondly, the retrospective nature of the study makes it difficult to include all factors likely to influence outcomes, such as intravascular imaging records. Thirdly, the evaluation was confined to in-hospital mortality and in-hospital MACEs without considering medium to long-term prognosis. Therefore, further large-scale prospective studies are necessary to ascertain whether delayed stenting can enhance therapeutic efficacy and financial benefits for STEMI patients following emergency thrombectomy.

## 5. Conclusions

In summary, concerning the timing of stent placement following emergency thrombus aspiration in STEMI patients, from the perspectives of clinical efficacy and hospitalization costs, the delayed stenting strategy was found to be non-inferior to the immediate stenting strategy and may potentially reduce myocardial injury. This finding suggests that delayed stenting is viable, permitting a more individualized surgical approach based on a meticulous assessment of thrombus burden and lesion complexity.

## Data Availability

The original contributions presented in the study are included in the article/supplementary material. Further inquiries can be directed to the corresponding author.

## Abbreviations

MI, myocardial infarction; STEMI, ST-segment elevation myocardial infarction; PCI, percutaneous coronary intervention; DE, distal embolization; MACEs, major adverse cardiovascular events; LDL-C, low-density lipoprotein cholesterol; Cr, creatinine; CRP, C-reactive protein; LVEF, left ventricular ejection fraction; BMI, body mass index; TIMI, thrombolysis in myocardial infarction; OR, odds ratio; CI, confidence interval.
